# Phenotypic Variability and Paternal Inheritance of a *CHD8* Variant Causing Intellectual Developmental Disorder With Autism and Macrocephaly Confirmed by Epigenetic and Structural Analyses

**DOI:** 10.1002/mgg3.70165

**Published:** 2025-12-17

**Authors:** Yutaka Furuta, Kimberly M. Ezell, Rizwan Hamid, Joy D. Cogan, Thomas A. Cassini, Lynette Rives, Ashley McMinn, Shailee Shah, Amanda C. Peltier, Stephen Layfield, Robin S. Fletcher, Matthew L. Tedder, Raymond J. Louie, Jennifer A. Lee, Jennifer Kerkhof, Jessica Rzasa, Bekim Sadikovic, Abdullah Al Mamun, Jonathan H. Sheehan, Christopher W. Moth, Jens Meiler, Marissa Vawter‐Lee, Paola Maria Mendoza‐Sengco, Jennifer B. Holzen, Sumit Pruthi, John A. Phillips, Rory J. Tinker, the Undiagnosed Diseases Network, Aaron Quinlan, Abdul Elkadri, Adeline Vanderver, Adriana Rebelo, Alan H. Beggs, Albert R. La Spada, Alden Huang, Alex Paul, Alexander Miller, Ali Al‐Beshri, Alistair Ward, Allen Bale, Allyn McConkie‐Rosell, Alyssa A. Tran, Andrea Gropman, Andres Vargas, Andrew B. Crouse, Andrew Stergachis, Anna Hurst, Anna Raper, Arjun Tarakad, Ashley Andrews, Ashley McMinn, Ashok Balasubramanyam, Barbara N. Pusey Swerdzewski, Beatriz Anguiano, Ben Afzali, Ben Solomon, Beth A. Martin, Bianca E. Russell, Brandon M. Wilk, Breanna Mitchell, Brendan C. Lanpher, Brendan H. Lee, Brent L. Fogel, Brett Bordini, Brett H. Graham, Brian Corner, Brianna Tucker, Bruce Korf, Calum A. MacRae, Camilo Toro, Cara Skraban, Carlos A. Bacino, Carol Oladele, Caroline Hendry, Carson A. Smith, Cecilia Esteves, Changrui Xiao, Chloe M. Reuter, Christine M. Eng, Chun‐Hung Chan, Colleen E. Wahl, Corrine K. Welt, Cynthia J. Tifft, Dana Kiley, Daniel J. Rader, Daniel Wegner, Danny Miller, Daryl A. Scott, Dave Viskochil, David A. Sweetser, David R. Adams, Deborah Barbouth, Deepak A. Rao, Devin Oglesbee, Devon Bonner, Donald Basel, Donna Novacic, Dustin Baldridge, Edward Behrens, Edwin K. Silverman, Elaine Seto, Elijah Kravets, Elisabeth Rosenthal, Elizabeth A. Worthey, Elizabeth A. Burke, Elizabeth Blue, Elizabeth C. Chao, Elizabeth L. Fieg, Ellen F. Macnamara, Elsa Balton, Emily Glanton, Emily Shelkowitz, Emily Wang, Eric Allenspach, Eric Klee, Eric Vilain, Erin Conboy, Erin E. Baldwin, Erin McRoy, Esteban C. Dell’Angelica, Euan A. Ashley, F. Sessions Cole, Filippo Pinto e Vairo, Frances High, Francesco Vetrini, Francis Rossignol, Francisco Bustos, Fuki M. Hisama, Gabor Marth, Gail P. Jarvik, Gary D. Clark, George Carvalho, Gerard T. Berry, Ghayda Mirzaa, Giorgio Sirugo, Gonench Kilich, Guney Bademci, Hector Rodrigo Mendez, Heidi Wood, Herman Taylor, Holly K. Tabor, Hongzheng Dai, Hsiao‐Tuan Chao, Hua Xu, Hugo J. Bellen, Hui Zhang, Ian Glass, Ian R. Lanza, Ingrid A. Holm, Isaac S. Kohane, Isum Ward, Ivan Chinn, J. Carl Pallais, Jacinda B. Sampson, James P. Orengo, James Verbsky, Jared Sninsky, Jason Hom, Jason Schend, Jennefer N. Kohler, Jennifer E. Posey, Jennifer Morgan, Jennifer Schymick, Jennifer Wambach, Jessica Douglas, Jiayu Fu, Jill A. Rosenfeld, Jimann Shin, Joan M. Stoler, Joanna M. Gonzalez, John A. Phillips, John Carey, John E. Gorzynski, John J. Mulvihill, Joie Davis, Jonathan A. Bernstein, Jordan Whitlock, Jose Abdenur, Joseph Loscalzo, Joy D. Cogan, Julian A. Martínez‐Agosto, Julie McCarrier, Justin Alvey, Kahlen Darr, Kaitlin Callaway, Kathleen A. Leppig, Kathleen Sullivan, Kathy Sisco, Kathyrn Singh, Katrina Dipple, Kayla M. Treat, Kelly Hassey, Kelly Schoch, Kevin S. Smith, Khurram Liaqat, Kim Worley, Kimberly Ezell, Kimberly LeBlanc, Kumarie Latchman, Lance H. Rodan, Laura Keehan, Laura Pace, Laurel A. Cobban, Lauren Blieden, Lauren C. Briere, Lauren Jeffries, Laurens Wiel, Layal F. Abi Farraj, Leoyklang Petcharet, LéShon Peart, Lili Mantcheva, Lilianna Solnica‐Krezel, Lindsay C. Burrage, Lindsay Mulvihill, Lisa Schimmenti, Lisa T. Emrick, Lorenzo Botto, Lorraine Potocki, Lynette Rives, Lynne A. Wolfe, Maija‐Rikka Steenari, Manish J. Butte, Margaret Delgado, María José Ortuño Romero, Maria T. Acosta, Marie Morimoto, Mariko Nakano‐Okuno, Mark Gerstein, Mark Wener, Marla Sabaii, Martha Horike‐Pyne, Martin G. Martin, Martin Rodriguez, Matt Velinder, Matthew Coggins, Matthew Might, Matthew T. Wheeler, MayChristine V. Malicdan, Megan Bell, Meghan C. Halley, Melissa Walker, Mia Levanto, Michael Bamshad, Michael F. Wangler, Michael Muriello, Michael Zimmermann, Michele Spencer‐Manzon, Miranda Leitheiser, Mohamad Mikati, Mohamad Saifeddine, Monika Weisz Hubshman, Monkol Lek, Monte Westerfield, Mustafa Tekin, Nada Derar, Naghmeh Dorrani, Neil H. Parker, Neil Hanchard, Nicholas Borja, Nicola Longo, Nicole M. Walley, Nitsuh K. Dargie, Odelya Kaufman, Oguz Kanca, Orpa Jean‐Marie, Page C. Goddard, Paolo Moretti, Patricia A. Ward, Patricia Dickson, Paul Berger, Paul G. Fisher, Pengfei Liu, Peter Byers, Pinar Bayrak‐Toydemir, Precilla D’Souza, Queenie Tan, Rachel A. Ungar, Rachel Li, Rachel Mahoney, Ramakrishnan Rajagopalan, Raquel L. Alvarez, Rebecca C. Spillmann, Rebecca Ganetzky, Rebecca Overbury, Rebekah Barrick, Richard A. Lewis, Richard L. Maas, Rizwan Hamid, Rong Mao, Ronit Marom, Rosario I. Corona, Runjun Kumar, Russell Butterfield, Sanaz Attaripour, Sandesh Nagamani, Sara Emami, Seema R. Lalani, Serena Neumann, Seth Perlman, Shamika Ketkar, Shamil R. Sunyaev, Shilpa N. Kobren, Shinya Yamamoto, Shrikant Mane, Shruti Marwaha, Sirisak Chanprasert, Stanley F. Nelson, Stephan Zuchner, Stephanie Bivona, Stephanie M. Ware, Stephen B. Montgomery, Stephen Pak, Steven Boyden, Suha Bachir, Surendra Dasari, Susan Korrick, Suzanne Sandmeyer, Tahseen Mozaffar, Tammi Skelton, Tanner D. Jensen, Tarun K. K. Mamidi, Taylor Beagle, Taylor Maurer, Teodoro Jerves Serrano, Terra R. Coakley, Thomas Cassini, Thomas J. Nicholas, Timothy Schedl, Tiphanie P. Vogel, Vaidehi Jobanputra, Valerie V. Maduro, Vandana Shashi, Vasilis Vasiliou, Virginia Sybert, Vishnu Cuddapah, Wendy Introne, Wendy Raskind, Willa Thorson, William A. Gahl, William E. Byrd, William J. Craigen, Winston Halstead, Yan Huang, Yigit Karasozen, Yong‐Hui Jiang

**Affiliations:** ^1^ Division of Medical Genetics and Genomic Medicine, Department of Pediatrics Vanderbilt University Medical Center Nashville Tennessee USA; ^2^ Department of Neurology Vanderbilt University Medical Center Nashville Tennessee USA; ^3^ Greenwood Genetic Center Greenwood South Carolina USA; ^4^ Molecular Genetics Laboratory, Division of Molecular Diagnostics London Health Sciences London Canada; ^5^ Department of Biomedical Data Science Meharry School of Applied Computational Sciences Nashville Tennessee USA; ^6^ Division of Infectious Diseases, Department of Internal Medicine Washington University School of Medicine St. Louis Missouri USA; ^7^ Department of Chemistry, Pharmacology, and Biomedical Informatics Center for Structural Biology and Institute of Chemical Biology, Vanderbilt University Nashville Tennessee USA; ^8^ Department of Pediatrics University of Cincinnati College of Medicine Cincinnati Ohio USA; ^9^ Division of Neurology Cincinnati Children's Hospital Medical Center Cincinnati Ohio USA; ^10^ Division of Pediatric Rehabilitation Medicine Cincinnati Children's Hospital Medical Center Cincinnati Ohio USA; ^11^ Tennessee Pediatrics Hendersonville Tennessee USA; ^12^ Department of Radiology Vanderbilt University Medical Center Nashville Tennessee USA; ^13^ Department of Medical Genetics and Genomics Icahn School of Medicine at Mount Sinai New York New York USA

**Keywords:** CHD8, epigenetics, intellectual developmental disorder with autism and macrocephaly (IDDAM), phenotypic variability

## Abstract

**Background:**

Intellectual developmental disorder with autism and macrocephaly (IDDAM, OMIM #615032) is an autosomal dominant neurodevelopmental disorder characterized primarily by intellectual disability, autism spectrum disorder, macrocephaly, tall stature, gastrointestinal symptoms, and variable neurological manifestations. Most cases result from de novo pathogenic variants in *CHD8*.

**Methods:**

We conducted genome sequencing through the Undiagnosed Diseases Network (UDN) in a female proband harboring a *CHD8* variant of uncertain significance (VUS), whose clinical presentation was consistent with IDDAM but included atypical features such as ptosis and hearing loss. Variant pathogenicity was further evaluated using EpiSign DNA methylation analysis and structural biology modeling.

**Results:**

Genome sequencing confirmed the *CHD8* variant inherited from her father, who exhibited a subtle feature, including traits consistent with attention‐deficit/hyperactivity disorder. Pathogenicity was confirmed through epigenetic signature testing (EpiSign), demonstrating characteristic methylation patterns and structural biology analysis, predicting significant protein destabilization.

**Conclusion:**

We describe the case of IDDAM caused by a paternally inherited *CHD8* variant. Our findings highlight the importance of considering parental inheritance in IDDAM diagnoses and suggest epigenetic and structural biology analyses as valuable tools for reclassifying VUS when variant pathogenicity remains uncertain.

## Graphical abstract

EpiSign (DNA methylation) analysis was performed on a proband and father with a CHD8 missense variant associated with IDDAM. Hierarchical clustering and multidimensional scaling showed that their methylation profiles closely matched individuals with a confirmed IDDAM episignature and were distinct from controls. The elevated MVP score further supported this similarity, indicating alignment with the IDDAM reference cohort.

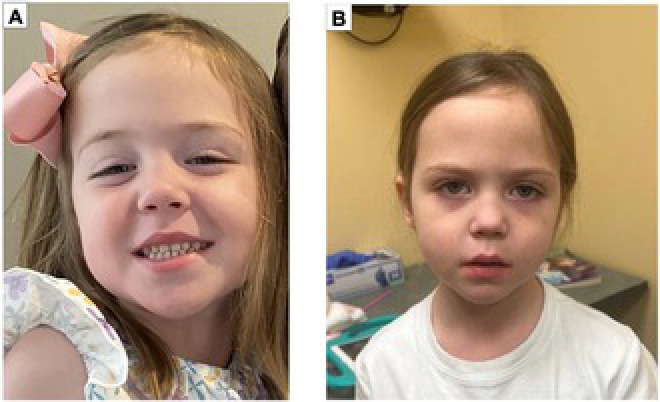

## 1 Introduction

Intellectual developmental disorder with autism and macrocephaly (IDDAM, OMIM #615032), also referred to as CHD8‐related neurodevelopmental disorder with overgrowth (CHD8‐NDD), is a condition with autosomal dominant inheritance characterized primarily by intellectual impairment, autism spectrum disorder (ASD), and macrocephaly (Douzgou et al. 2019; Mitchel et al. 2022; Ostrowski et al. 2019). Affected individuals commonly exhibit additional features, including tall stature, gastrointestinal symptoms, neurological abnormalities, and distinct facial features. Approximately 80% develop macrocephaly during infancy and tall stature during puberty. ASD and developmental delays occur in more than 75% of affected individuals, while attention‐deficit/hyperactivity disorder (ADHD) occurs in about half (Hanly et al. 2021; Mitchel et al. 2022). Gastrointestinal symptoms, particularly constipation, are present in 63%. Neurologic symptoms include hypotonia (30%), seizures (10%–15%), dystonia (rare), and Chiari I malformation (rare) (Mitchel et al. 2022).

IDDAM typically arises due to a de novo heterozygous pathogenic variant in *CHD8*, though rare maternally inherited cases from cognitively unaffected individuals have been reported (Ostrowski et al. 2019). We report here a novel and unusual case of a child whose clinical phenotype aligns strongly with IDDAM, caused by a paternally inherited *CHD8* variant initially classified as a variant of uncertain significance (VUS). The pathogenicity of this variant was confirmed through epigenetic signature testing, demonstrating characteristic methylation changes, and structural biology analysis, predicting protein destabilization. This report emphasizes the importance of considering parental inheritance in IDDAM diagnoses and suggests that epigenetic and structural biology analyses can be valuable tools for reclassifying VUS when pathogenicity remains unclear.

## 2 Case Report

Our proband is a 5‐year‐old female born prematurely at 34 weeks and 3 days gestation via cesarean section, following a pregnancy complicated by maternal type 1 diabetes mellitus, partial placental abruption, and preeclampsia. Neonatal complications included respiratory insufficiency and feeding difficulties, necessitating a 3‐week NICU admission.

**FIGURE 1:**
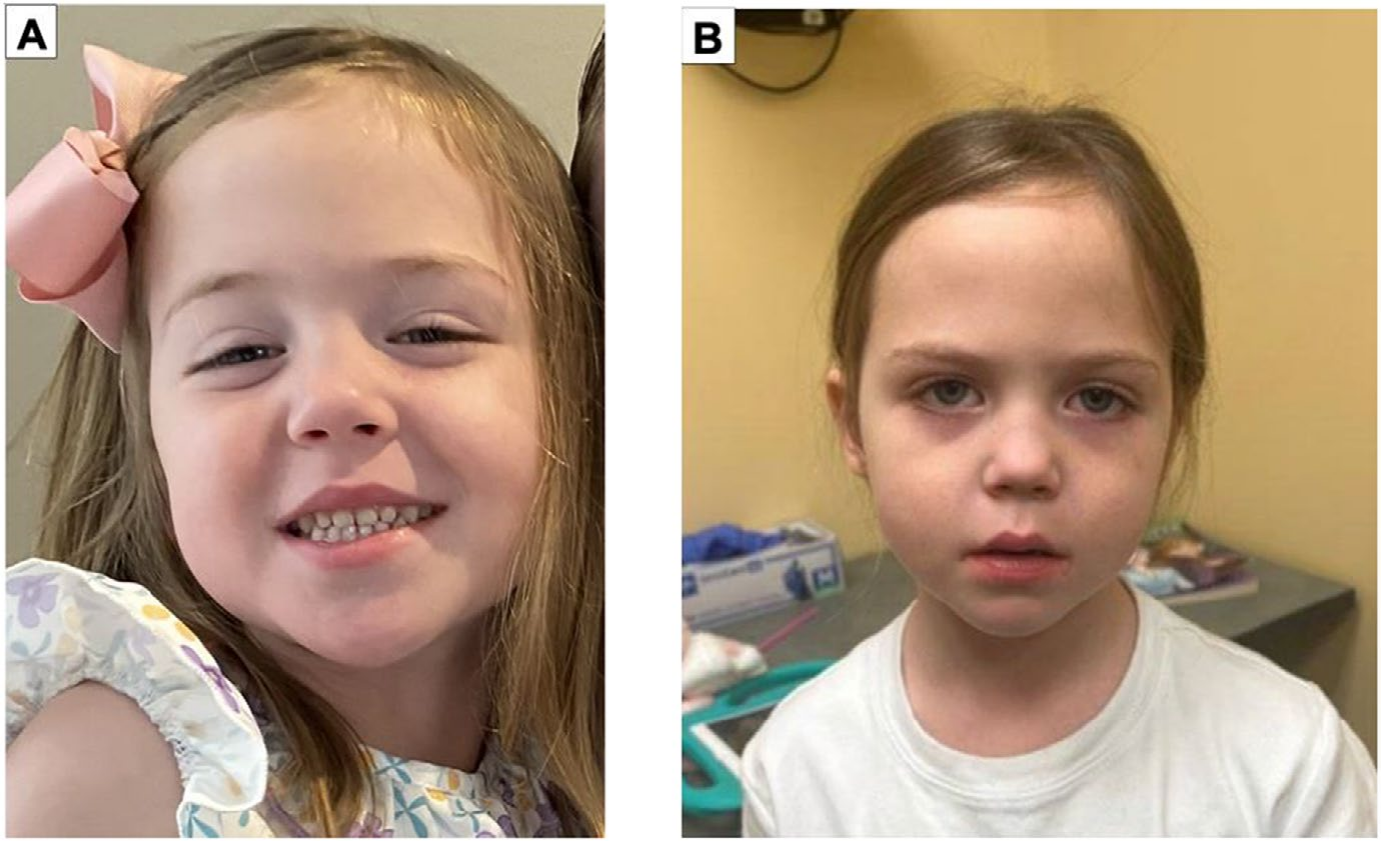
(A) Ptosis before use of pyridostigmine. (B) Improved ptosis after use of pyridostigmine.

At 6 months, hypotonia was noted, prompting neurological and genetic evaluations that were initially inconclusive. By 18 months, hypotonia had worsened significantly, raising concerns for hypotonic cerebral palsy. Progressive feeding difficulties led to failure to thrive, prompting placement of a gastrojejunostomy (GJ) tube. At Age 2, she developed seizures of several semiologies requiring trials of multiple anti‐seizure medications as well as evolving distal ankle spasticity and dystonia requiring orthotics and antispasmodic medications. At 2.5 years, progressive ptosis impaired daily activities (Figure 1A). Clinical suspicion for myasthenia gravis was supported by partial improvement with pyridostigmine (Figure 1B), despite negative autoimmune and congenital myasthenia panels and inconclusive electromyography (EMG). EMG at 3 years old was abnormal with decrement of her abductor pollicis brevis muscle to repetitive nerve stim; all other muscles were normal with no decrement. EMG repeated at a later age again showed decrement, this time present in 4/4 muscles tested, with 2/4 showing significant decrement. She also exhibited intermittent dystonia responsive to carbidopa‐levodopa therapy and right‐sided sensorineural hearing loss managed with a hearing aid. She had severe constipation associated with fecal incontinence requiring extensive evaluation by gastroenterology and multiple procedures, with eventual ileostomy. She later developed concerns for neurogenic bladder as well.

She demonstrated mild delays in achieving the following developmental milestones, including: smiling at 3 months, rolling at 6 months, sitting independently at 12 months, standing at 18 months, walking independently at 22 months, and speaking first words around 2.5 years. Then, she was diagnosed with autism spectrum disorder.

Family history revealed bilateral hearing loss in her mother (onset at 29 years), right‐sided hearing loss in her maternal uncle (onset in 30s), and hearing loss in her maternal grandmother (onset in 50s). Her father's head circumference was in the 93rd percentile, and he had tall stature (185 cm) and traits suggestive of ADHD, without a formal diagnosis of developmental or intellectual disabilities.

Physical examination revealed macrocephaly (> 98th percentile), tall stature (> 98th percentile), pointed chin, ptosis, mild scoliosis, lordosis, antigravity strength throughout with weakness over both hand grip and active ankle movements, mild spasticity over both ankles, and abnormal gait characterized by foot slap and midfoot overpronation on stance. Facial photos are shown in Figure 1.

Chromosomal microarray and initial brain MRI were normal. Myasthenia gravis antibody labs were negative. Initial proband‐only exome sequencing at 15 months identified a variant of uncertain significance (VUS), c.3533G>A (p.Arg1178His), in *CHD8* (NM_001170629.2). This variant had a Combined Annotation‐Dependent Depletion (CADD) score of 28.5 and a Genomic Evolutionary Rate Profiling (GERP) score of 6.54, and it was not present in gnomAD (v2.1.1). A congenital myasthenia gene panel identified a heterozygous c.4306C>T (p.Arg1436Cys) VUS in *LAMB2* (NM_002292.3), later bioinformatically determined as likely benign and clinically insignificant. A repeat brain MRI at 4 years old showed the corpus callosum was mildly dysmorphic.

Due to diagnostic uncertainty, the patient was referred to the Undiagnosed Diseases Network (UDN) at Vanderbilt. Reanalysis through genome sequencing aimed to clarify the inheritance of the *CHD8* variant, identify alternative causative variants, and investigate a genetic basis for her familial hearing loss. Genome sequencing confirmed the *CHD8* variant was paternally inherited, with no additional pathogenic variants identified. While most clinical features strongly aligned with IDDAM (OMIM #615032), ptosis and hearing loss were notably atypical.

## 3 Methods and Results

### 3.1 DNA Methylation Data Analysis Methods and Results

To investigate the possibility of underlying epigenetic change in *CHD8*, methylation analysis was conducted using the clinically validated EpiSign assay through Greenwood Genetics, following previously established methods (Sadikovic et al. 2021; Aref‐Eshghi et al. 2020; Levy et al. 2021; Kerkhof et al. 2024). Methylated and unmethylated signal intensities generated from the EPIC V2 arrays were imported into R 4.2.1 for normalization, background correction, and filtering. Beta values were then calculated as a measure of methylation level, ranging from 0 (no methylation) to 1 (complete methylation), and processed through the established support vector machine (SVM) classification algorithm for EpiSign disorders. The classifier utilized the EpiSign Knowledge Database, which consists of over 20,000 methylation profiles from reference disorder‐specific and unaffected control cohorts, to generate disorder‐specific methylation variant pathogenicity (MVP) scores. These MVP scores are a measure of prediction confidence for each disorder and range from 0 (discordant) to 1 (highly concordant). A positive classification typically generates MVP scores > 0.5. The final matched EpiSign result is generated using these scores, along with the assessment of hierarchical clustering and multidimensional scaling.

**FIGURE 2:**
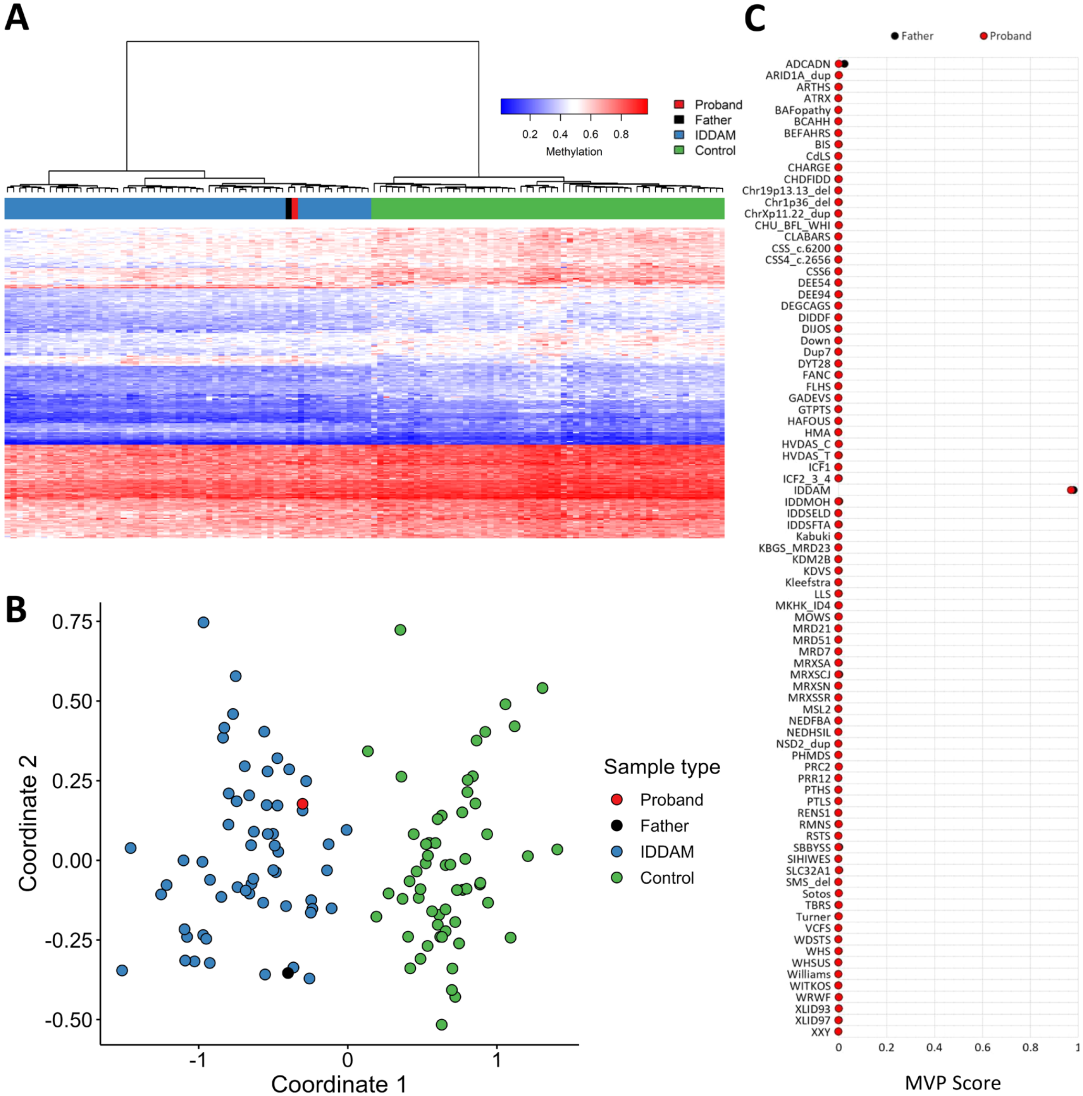
EpiSign (DNA methylation) analysis of peripheral blood from a proband and father with a missense variant in *CHD8*, the causative gene for IDDAM. (A) Hierarchical clustering and (B) multidimensional scaling plots indicate the proband (red) and father (black) have a DNA methylation profile similar to subjects with a confirmed IDDAM episignature (blue) and distinct from controls (green). (C) MVP score, a multi‐class supervised classification system capable of discerning between multiple episignatures by generating a probability score for each episignature. The elevated score for IDDAM shows an episignature similar to the intellectual developmental disorder with autism and macrocephaly reference cohort.

EpiSign variant targeted analysis revealed a genome‐wide DNA methylation profile consistent with IDDAM for both proband and father (Figure 2). EpiSign analysis was concordant with a methylation signature observed in patients with *CHD8* variants, as indicated by Euclidean clustering, multidimensional scaling, and an elevated MVP score in both the proband (MVP = 0.971) and father (MVP = 0.981).

### 3.2 Structural Biology Analysis Methods and Results

The Chromodomain‐Helicase‐DNA‐binding protein 8 (CHD8) is an ATP‐dependent DNA helicase that acts as a chromatin remodeling factor and regulates transcription and splicing of genes involved in neuronal differentiation, cell cycle, and DNA repair, including beta‐catenin, STAT3, p53, and WNT signaling pathways (Thompson et al. 2008). It contains 2581 amino acids organized into two CHROMO (CHRromatin Organization MOdifier) domains, a SNF2‐related/DEXDc (DEAD‐like helicases superfamily) domain, a C‐terminal helicase domain of the SNF family helicases, a Homeodomain‐like domain, and two BRK domains. The Arg1178His *CHD8* variant was evaluated using the VUStruct (Moth et al. 2024) web resource for personalized structural biology and judged to be likely deleterious.

**FIGURE 3:**
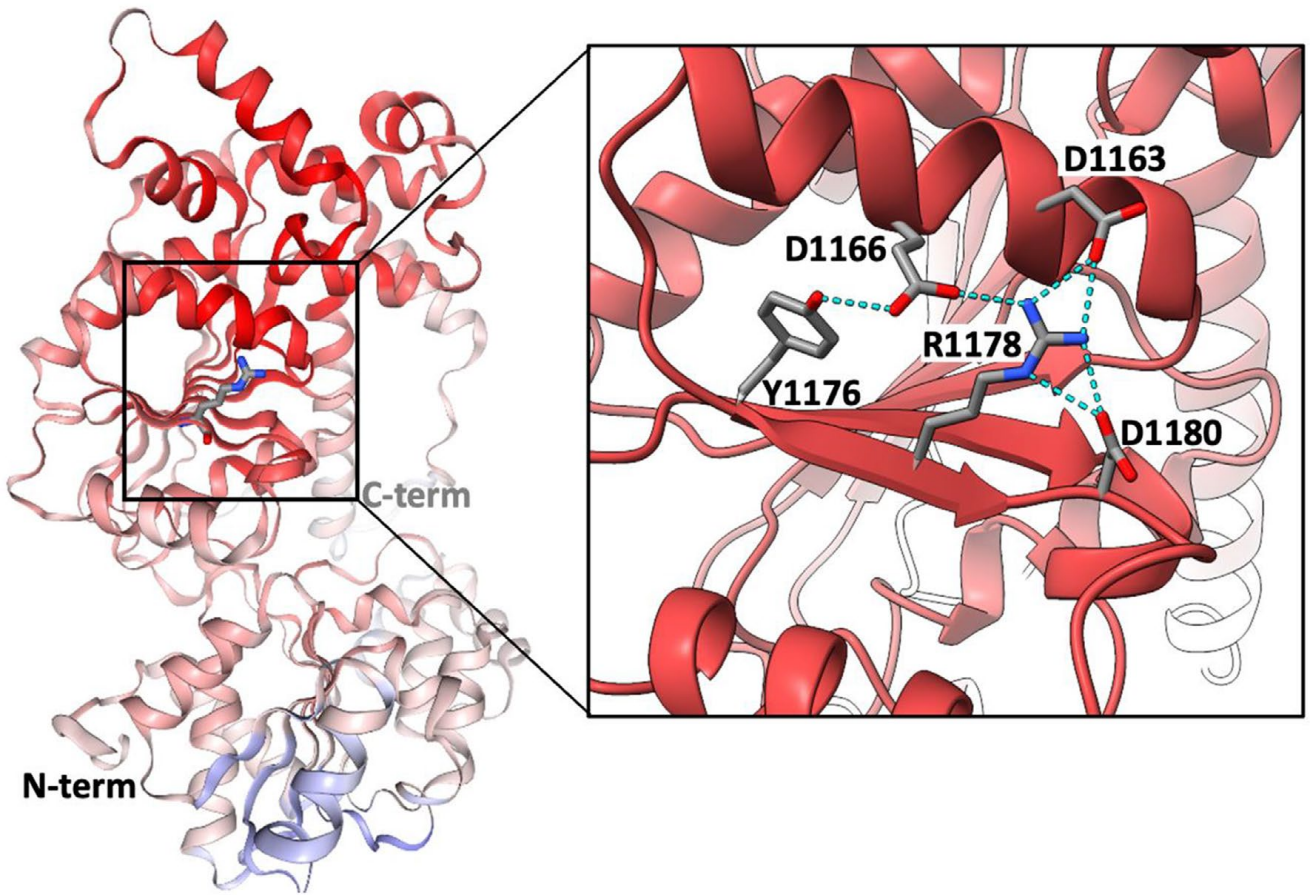
Structural model of CHD8, Chromodomain‐Helicase‐DNA‐binding protein 8 (residues 801–1350), showing location of the R1178H mutation. Backbone is colored according to 3D colocalization of deleterious variants (Pathprox value most pathogenic, deep red, to least pathogenic, blue). Inset shows the predicted H‐bond network (cyan) stabilizing the tertiary structure of the helicase domain, which would be disrupted by the *R*>H substitution (Rosetta ddG score > 5 REU). Model is AF‐Q9HCK8‐F1‐v4 from AlphaFold Database.

Only the structure of the BRK domain (residues 2309–2372) has been determined experimentally (Ab et al. 2006), so the analysis was performed on five comparative models from the SWISS‐MODEL (Bienert et al. 2017) database and one model from the AlphaFold (Varadi et al. 2022) database, all of which included position 1178 in the DEAD‐like helicase domain. The side chain of Arg1178 is predicted to form a hydrogen‐bonding network with four neighboring residues (D1163, D1166, Y1176, D1180) on the interface between a central beta‐pleated sheet and a nearby alpha‐helix (Figure 3) which stabilizes the tertiary structure of the domain. The *R*>H substitution disrupts this network, and a Rosetta ddG Cartesian calculation (Frenz et al. 2020; Park et al. 2016) predicts that it destabilizes the protein by an average of 6.5 REU (Rosetta Energy Units, which scale approximately with kcal/mol). To test for a 3D colocalization of deleterious mutations, the PathProx algorithm (Sivley et al. 2018) was used to map all 13 pathogenic and likely pathogenic variants from ClinVar onto the models, and compare the distribution with that of 193 putative neutral variants from gnomAD. Values ranged from 0 to +0.4, suggesting weakly that the disease‐associated variants do cluster in space more than the benign variants, and the Arg1178His variant falls in a 3D hot spot. This result agrees with the COSMIS *z*‐score of −2.23, indicating that the spatial context of that position is significantly intolerant to missense mutations (Li et al. 2022). This is likewise consistent with the AlphaMissense (Tordai et al. 2024) score of 0.993 indicating likely pathogenic. Moreover, our analysis agrees well with previous modeling studies of variants in CHD8 protein (An et al. 2020). Figure 3 was made with ChimeraX (https://www.cgl.ucsf.edu/chimera/). Taken together, the results of these calculations are consistent with a loss‐of‐function effect due to destabilization of the protein structure.

## 4 Discussion

Here we describe the case of IDDAM caused by a paternally inherited *CHD8* variant, initially classified as a VUS. This diagnosis was confirmed through epigenetic testing, which identified characteristic DNA methylation changes consistent with IDDAM, and structural biology analysis, which predicted significant destabilization of the CHD8 protein product.

Although IDDAM typically results from de novo *CHD8* variants, parental inheritance is also possible. Ostrowski et al. previously reported maternal inheritance in approximately 11% of their studied cases but did not observe paternal inheritance (Ostrowski et al. 2019). However, a case of autism spectrum disorder with a paternally inherited *CHD8* variant (p.Arg1797Gln) has been reported (Bernier et al. 2014). Most recently, Siu et al. reported siblings with autism spectrum disorder harboring a paternally inherited *CHD8* variant, which was verified for the first time by episignature analysis (Siu et al. 2019). Substantial clinical variability associated with *CHD8* variants was previously described (Dingemans et al. 2022). Although clear genotype–phenotype correlations have not been established, individuals with a missense *CHD8* variant were less severely affected than those with other variant types such as nonsense, frameshift, or splice‐site variants (Dingemans et al. 2022; Mitchel et al. 2022). The pathogenicity of our patient's missense *CHD8* variant was supported by epigenetic testing and structural biology analysis. Our case uniquely demonstrates paternal inheritance, highlighting an important expansion of the known inheritance spectrum of IDDAM. The father exhibited a phenotype limited to ADHD‐like tendencies, which are relatively common in the general population. Given his proportionate head circumference and absence of developmental delays or intellectual disabilities, it remains uncertain whether variable expressivity is present in our case, although it cannot be excluded. Therefore, until more data become available, we suggest that a high index of suspicion for inherited *CHD8* variants is warranted, even when parental clinical features are mild, atypical, or absent.

Our proband presented two atypical clinical features: ptosis and hearing loss, neither commonly associated with IDDAM nor listed in the OMIM phenotypic synopsis (Table 1). Ptosis, responsive to pyridostigmine, raised the clinical suspicion of a neuromuscular junction disorder. Notably, Lee et al. reported a similar association of a spontaneous heterozygous *CHD8* variant with myasthenia‐like features (Lee et al. 2020), suggesting a potential but still poorly understood role of CHD8 in neuromuscular junction integrity or function. In contrast, our proband's hearing loss is likely not to be related to the *CHD8* variant, given the distinct maternal family history and its segregation pattern, suggesting a distinct genetic etiology. However, no other variants that could contribute to the hearing loss or ptosis were identified on her genome sequencing.

**TABLE 1:** Comparison of clinical features reported in OMIM #615032 and our proband.

OMIM #615032 reported features	Our proband
Tall stature	+
Macrocephaly	+
Supraorbital ridge	−
Downslanting palpebral fissures	−
Gastrointestinal problems	+
Constipation	+
Autism	+
Seizures	+
Regression	−
Sleeping disturbances	+

In conclusion, clinicians should maintain a high index of suspicion for inherited *CHD8* variants even when parental clinical features are mild, atypical, or not present at all. Epigenetic testing and structural biological modeling can be useful in reclassifying VUS when the significance of a *CHD8* variant is uncertain. This case highlights the necessity of integrating multiple lines of evidence, including clinical, genetic, protein modeling, and epigenetic data, in individuals presenting with features of IDDAM to make a diagnosis.

## Author Contributions

Y.F. and R.J.T. drafted the article. Y.F., R.J.T., R.H., J.D.C., T.A.C., L.R., J.A.P., R.S.F., M.L.T., R.J.L., J.A.L., J.K., J.R., B.S., A.A.M., J.H.S., C.W.M., and J.M. were responsible for the correct description, display, and interpretation of the genomic diagnostic and technical parts. Y.F., R.S.F., M.L.T., R.J.L., J.A.L., J.K., J.R., B.S., A.A.M., J.H.S., C.W.M., and J.M. created the table and figures. R.J.T., J.A.P., T.A.C., R.S.F., M.L.T., R.J.L., J.A.L., J.K., J.R., B.S., A.A.M., J.H.S., C.W.M., J.M., M.V.‐L., and P.M.M.‐S. helped to draft the article. All authors read, corrected, and approved the final article.

## Funding

This work was supported in part by the National Institutes of Health (NIH) Common Fund, National Human Genome Research Institute (NHGRI) grant 15‐HG‐0130 (J.A.P., R.H., J.D.C.). This work was funded in part through support from the Potocsnak Center for Undiagnosed and Rare Disorders.

## Disclosure

Molecular graphics and analyses performed with UCSF ChimeraX, developed by the Resource for Biocomputing, Visualization, and Informatics at the University of California, San Francisco, with support from National Institutes of Health R01‐GM129325 and the Office of Cyber Infrastructure and Computational Biology, National Institute of Allergy and Infectious Diseases. https://www.cgl.ucsf.edu/chimera/.

## Ethics Statement

This study was approved by the Undiagnosed Diseases Network (UDN) Publications and Research Committee and was performed in accordance with the Declaration of Helsinki.

## Consent

Written informed consent for publication, including the use of clinical photographs, was obtained from the patient's parents.

## Conflicts of Interest

The authors declare no conflicts of interest.

## Data Availability

Data sharing is not applicable to this article as no new data were created or analyzed in this study.
